# Reflecting the voices of prison officers with respect to their support, supervision, and wellbeing training needs: a reflexive thematic analysis

**DOI:** 10.3389/fpubh.2025.1656223

**Published:** 2025-09-22

**Authors:** Joanne Forsyth, Jennifer Shaw, Andrew Shepherd

**Affiliations:** Faculty of Biology, Medicine and Health. Division of Psychology and Mental Health, School of Health Sciences, The University of Manchester, Manchester, United Kingdom

**Keywords:** prison officer wellbeing, prison officer support, prison officer supervision, prison cultures, prison officer culture, emotional labour

## Abstract

Prisons represent a significant public health concern. The challenging living and working conditions within prisons are widely acknowledged to contribute to elevated rates of ill health among both prisoners and prison officers. Officers hold a vital role in supporting individuals in custody. However, the intense pressures associated with the role, compounded by toxic workplace cultures, are frequently reported to negatively impact their wellbeing. This deterioration not only affects officers personally but can also compromise the effective functioning of the prison service. While structured support systems exist to meet the rehabilitation and care needs of prisoners, equivalent care and professional support for prison officers remains inadequate and insufficiently prioritised. A total of 27 in-depth qualitative interviews were conducted with both former and current prison officers to explore their subjective experiences and to collaboratively identify the support, supervision, and wellbeing training needs arising from their roles. A process of reflexive thematic analysis was adopted. Six main themes were constructed from the data analysis: (1) responsible recruitment, training, and development; (2) dual duty of care; (3) acknowledgement of psychological hardship; (4) superficial support systems; (5) collaborative cultural change; and (6) components of a good model of practice. This study highlights the urgent need for reform in how prison officers are supported and serves as a framework for the development of more effective support structures. It also contributes to the growing body of literature by deepening our understanding of the emotional labour inherent in the role and the associated psychological impact. Furthermore, it acknowledges the wider societal implications of these findings, emphasising that supporting prison officers is a matter of institutional responsibility and a critical public health concern.

## Introduction

Prisons represent a significant public health concern due to the complex social, psychological, and physical challenges they present. It is widely acknowledged that prisons are often unhealthy, unsafe, and potentially harmful environments, affecting the wellbeing of both those in custody and those who work within them (1). As of March 2024, the prison population in England and Wales was approximately 87,900, with projections estimating an increase to between 95,100 and 114,200 by 2027 (2). In England and Wales, the lack of significant investment and the limited construction of new prison facilities has resulted in widespread overcrowding, with many institutions operating beyond their designed capacity (2). Overcrowding has a profound impact on individuals living and working within the criminal justice system as well as society at large (3). Severe overcrowding contributes to increased violence, exacerbates physical and mental health issues, for both prisoners and staff, impeding efforts to rehabilitate offenders and potentially leading to reoffending (4). This highlights the urgent need for systemic reform that prioritises health, safety, and rehabilitation within the criminal justice system.

Prisons are also chronically understaffed intensifying existing challenges, placing further strains on prison officers, contributing to their overall stress, often leading to burnout, and ill health (5). Currently, there is no mandated national ratio of prison officers to prisoners, which may be due to the varying operational needs and security levels across different prisons. In June 2023, the total prison population in the UK was approximately 95,526 people, encompassing 85,851 in England and Wales, 7,775 in Scotland, and 1,900 in Northern Ireland (6) with the full time equivalent of 22,426 prison officers in post looking after these prisoners (7). The systemic understaffing of prisons not only jeopardises staff wellbeing but also highlights broader organisational shortcomings in workforce planning and institutional care.

In England and Wales, prisons are public institutions, whether operated directly by the government or privately under government contracts, they are funded by taxpayers. Therefore, there is a societal interest in how they function, how those incarcerated are treated, and how staff are supported. Prisons serve multiple purposes. In the UK, these include retribution, incapacitation, deterrence, and rehabilitation, within a framework that aims to be just, humane, and effective (8). How and whether these purposes are fulfilled has significant implications not only for prisoners but also for the officers responsible for looking after them, highlighting the importance of evaluating whether current practices genuinely align with the prison service’s stated aims, both in principle and in practice.

The ‘pains of imprisonment’ refer to the subjective experiences of hardship and deprivation endured by prisoners (9, 10). However, recent research has expanded this lens to include the ‘pains of being a prison officer’ (11), recognising that prison officers are also subjected to institutional and psychological strain. This invites a more holistic understanding of the prison environment investigating how harm is experienced across its workforce as well as the prisoner population. Theoretically, these strains can be understood through Hochschild (12) concept of emotional labour, which highlights how organisations require individuals to regulate and perform emotions in ways that align with institutional norms and expectations.

Emotional labour, in the context of prison officers, reveals the often overlooked psychological and relational demands of their work, demands that go far beyond the physical and procedural aspects typically associated with the role (13). Prison officers hold a vital role in supporting prisoners during their time in custody and assisting in their rehabilitation. The role of the modern prison officer is multifaceted, complex, and challenging (14). Research has demonstrated that staff wellbeing directly influences the quality of care and support they are able to provide (15–17). The prevalence of mental illness, substance misuse, and infectious diseases is at least twice as high among prisoners compared to the general population (75), highlighting the complex and intensive needs of this group. These findings highlight both the moral and practical imperative to adequately support not only prisoners but also the prison officers who look after them.

Officers must rely on their ‘jail craft’, the practical knowledge and interpersonal skills developed through experience, not only to maintain order and security, but also to support prisoners and contribute to their rehabilitation (13, 18). The different approaches taken by officers can set the tone, or culture, on a prison wing and influence relationships both with and among prisoners and colleagues. Striking the right balance between becoming too involved or too detached is a challenging skill to master (19) and requires continuous emotional regulation, aligning closely with Hochschild (12) theory of emotional labour. As officers navigate their occupational and organisational environments, these paradoxical duties and conflicting responsibilities can become a significant source of stress (20). Without sufficient training, support, and supervision, the capacity of prison officers to manage the emotional demands of their role can be undermined (21). This not only exacerbates the psychological toll of emotional labour but can also compromise their professional efficacy and resilience, reinforcing a cycle of occupational strain and institutional dysfunction.

Within the prison context, emotional labour is intensified by officers’ exposure to traumatic events, such as supporting prisoners at risk of self-harm or suicide, responding to deaths in custody, and navigating episodes of violence, where officers are required to maintain professional composure and authority and suppress personal distress (13, 22). Exposure to traumatic events can also contribute to burnout, compassion fatigue, and reduced compassion satisfaction (23). Furthermore, repeated exposure to violence and traumatic incidents is associated with a range of significant psychological difficulties, including anxiety, depression, and post-traumatic stress disorder (PTSD) (24). Repeated exposure to violence and traumatic incidents is associated with a range of significant psychological difficulties, including anxiety, depression, and post-traumatic stress disorder (PTSD) (25).

Prolonged occupational stress, intensified by heavy workloads, long working hours, and role conflict, where officers must balance security with care (19, 78), can contribute to emotional exhaustion, burnout, low morale, reduced job satisfaction, absenteeism, and high staff turnover (26, 28). These outcomes highlight the need for systemic support structures within the prison service to safeguard the wellbeing of prison officers. Furthermore, organisational stressors such as working in environments marked by excessive noise, overcrowding, unsanitary conditions, and deteriorating infrastructure, compound the psychological toll on prison officers (24). These adverse conditions not only impede daily functioning but also amplify the emotional labour required to perform roles that demand composure, authority, and emotional restraint (12). These challenges have been further intensified by the ongoing recruitment and retention crisis within the prison service in England and Wales leading to chronic understaffing (30) which places additional demands on already over stretched prison officers. The cumulative impact of these stressors often extends beyond the prison walls, resulting in a deterioration in officer wellbeing and work-life conflict (31). Despite these known risks, employing bodies continue to fall short of meeting the minimum standards for psychological health and safety as outlined by the UK Health and Safety Executive (32, 33). Such systemic shortfalls raise serious concerns regarding the duty of care toward prison officers and highlight the urgent need for meaningful interventions to support the emotional and psychological wellbeing of prison officers.

Fundamentally, the emotional labour performed by prison officers is intrinsically linked to the culture of the prison, shaping, and being shaped by the interpersonal dynamics, expectations, and values within the institution (34). These dynamics raise important questions concerning organisational responsibility and the ethics of care within prison environments. Prison officers have frequently described a need to present and uphold an image of silence, bravado, and machismo (35, 36), only expressing emotion in the ‘right’ circumstances, often managing their own emotions and the emotions of prisoners simultaneously (13). Within this culture, it is often perceived as a sign of weakness to show emotion or seek support (14) which reinforces a cycle of emotional suppression. This is further exacerbated by the presence of toxic masculinity, which intensifies the emotional demands required of prison officers, which may lead them to adopt a façade of coping while suppressing genuine emotional responses (37). Nylander and Bruhn (34) highlight two forms of emotional labour in this context, surface acting and deep acting, both of which sustain institutional norms while masking the psychological costs. The cumulative effect of this emotional labour, shaped by institutional norms, risks leaving officers’ support needs going unnoticed and unsupported.

Cultures provide us with intellectual, emotional, and physical knowledge to understand our lives (18). The distinction between organisational culture (top-down) and occupational culture (bottom-up) has been recognised (18) with a dialectic between the two (38). Organisational cultures directly impact upon staff wellbeing, influencing burnout, retention, and recruitment which are fundamental to the future of organisations and their capacity and capability to provide a service (17). Occupational cultures can be understood as socially constructed and internalised matrices of shared thinking, feeling, and behaving (38). Cultures shape working practices and individuals’ sense of self (18), while also providing cultural stability within organisations (76). Therefore, a reciprocal relationship exists between the occupational identities of individuals and groups, as they are both shaped by and contribute to the organisational and occupational cultures in which they are embedded (39). Thus, inevitably, prison officers are intrinsically influenced by the prison cultures they are immersed within. Ultimately, a positive and supportive culture promotes collaboration, continuous and sustained learning, and encourages staff resilience, whereas a negative culture can lead to burnout, inefficiency, and compromised care (40). Therefore, recognising and actively shaping prison cultures is essential for safeguarding officer wellbeing, and ensuring prison environments function effectively, ethically, and sustainably. Furthermore, given the well-documented psychological impact of exposure to traumatic events on prison officer wellbeing, it is imperative to challenge embedded toxic cultural norms that suppress emotional expression (36). Such toxic norms not only reinforce the emotional burdens placed on officers but also inhibit access to meaningful support and perpetuate a culture of toxic masculinity within prison settings (41). Promoting healthier approaches to emotional expression, fostering psychological safety, and ensuring access to structured support and supervision are essential steps toward improving officer wellbeing and creating a more humane, resilient, and sustainable prison environment.

A systematic review of the literature, investigating the support and supervision needs of prison officers, was reported as an empty systematic review as it found no evidence that met the inclusion and exclusion criteria and the protocol registered with PROSPERO (21). The review identified some examples of formal supervision practices in specialist prison environments such as Therapeutic Communities (TCs), Psychologically Informed Planned Environments (PIPEs), and the Unlocked Graduates (UG) scheme. Supervision should be central to the development of prison officers’ knowledge and skills, providing a structured space for reflection, learning, and professional growth (42). The integration of supervision and support alongside enhanced psychological mindedness has been shown to positively influence interpersonal relationships, prison culture, and the wider prison environment (43). However, despite its recognised importance, there is no evidence in the literature that formal supervision is routinely available to all prison officers (21). Wellbeing training, which includes structured interventions designed to enhance awareness, resilience, and coping strategies for managing stress, has been shown to reduce distress and promote wellbeing (44) making it particularly appropriate in this context. Building on this evidence, the present study engaged with current and former prison officers to explore their support, supervision, and wellbeing training needs, with the aim of informing the future development of a comprehensive model of care.

## Methods

### Design

This study employed a qualitative design, utilising semi-structured interviews with current and former prison officers to explore their subjective experiences and collaboratively identify the support, supervision, and wellbeing training needs associated with their roles.

### Procedure

Initial contact was made with a senior member of the Prison Officers’ Association (POA), who disseminated the research information to the POA National Executive Committee. Upon receiving approval, an information flyer was circulated to prison officers through POA communication channels. Interested officers contacted the researchers directly for further information.

A semi-structured interview approach was adopted, utilising open-ended questions from a prepared list. This list was piloted with a multidisciplinary peer supervision group of professionals to ensure clarity, readability, and logical flow. The group was briefed on the study’s purpose and aims, and no concerns were raised. While core interview questions remained consistent throughout the study, additional questions were asked as needed to allow flexible, in-depth exploration of relevant phenomena (45). All interviews were conducted online via Zoom.

### Participants

A purposive sampling strategy was used to recruit individuals who were knowledgeable about the phenomenon under investigation (46), and who could articulate their experiences and perspectives in depth (47). All participants had worked, or were currently working, in prison establishments in England. All contributions were considered valid and valuable in shaping a comprehensive understanding of prison officers’ support, supervision, and wellbeing training needs. Therefore, no inclusion or exclusion criteria were applied based on length of service, time since leaving the service, or prison category, in order to capture a broad range of experiences and perspectives. Reaching saturation was not an aim of this study, as the concept is not aligned with the chosen analytical approach of Reflexive Thematic Analysis (RTA), where theme development is viewed as an interpretative and iterative process (48). Consequently, interviews were undertaken over an extended period between May 2021 and June 2022. A total of 27 interviews were conducted, generating 47 hours and 32 minutes of recorded data. Participants’ length of service ranged from 18 months to 45 years, with a mean of 16.56 years. Of the 27 participants, 20 identified as male and 7 as female, 14 were currently serving prison officers, including one on the Unlocked Graduates scheme, and 13 were former officers. All participants identified as White.

### Data analysis

The interviews were conducted, transcribed, and initially analysed by JF. Identified themes were discussed collaboratively between JF, JS, and AS. Subsequently, JF produced a written analysis, on which JS and AS provided independent feedback. The final analysis was completed by JF.

The interviews were analysed thematically following a six-phase process (49). Although the six phases are outlined in a successive order they were used as a flexible set of guidelines, the analysis was not a linear process. Initial analysis began with the first 11 interviews with former prison officers. These preliminary findings were presented at a research seminar to gather feedback and enhance analytical rigour. As no changes to the interview questions were deemed necessary, interviews with an additional two former officers and 14 current serving officers were conducted and analysed consecutively.

The principles of RTA were adopted whereby the researcher embraced an active role in knowledge production (50). The analysis involved an inductive and recursive approach through continual engagement with the data to identify meaningful patterns. Repeated cycles of reading, coding and theme development were undertaken. Codes represented single ideas associated with a segment of the data and served as building blocks to the themes, which reflected the researcher’s interpretive understanding of meaning across the data set. The process was intentionally unstructured and organic, as opposed to employing a specific coding framework, and the generation of themes was the outcome of the entire process (48). While the RTA approach does not rely on saturation as a benchmark, it was observed that toward the later interviews, participants were no longer introducing new themes or insights.

### Reflexivity

Reflexivity is a core component of RTA; therefore, it was necessary for the researchers to remain open about their potential influences and assumptions throughout the research process (50). This analysis aligns with the researchers’ epistemological position of pragmatism, which prioritises the research question over adherence to any single methodological or philosophical stance (46). While positionality concerns what we know and believe, reflexivity refers to how we critically engage with and apply that knowledge during the research process (51).

The researchers engaged in ongoing reflexive practice, critically examining their assumptions, beliefs, and potential biases, and considered how these may have shaped the research. They acknowledged a shared professional interest in prison environments and staff wellbeing, alongside a belief in the value of appropriate support, supervision, and training, particularly in the context of such a demanding role.

To support this process, JF maintained a reflexive journal, documenting her reflections, decisions, and emotional responses during data collection and analysis. This practice offered a space to track the evolution of her thoughts and perspectives, and enhanced transparency by highlighting how her assumptions and experiences informed interpretation. The journal also captured her emotional responses to participants’ accounts, the relational dynamics during interviews, and the challenges encountered, fostering greater self-awareness, and contributing to ongoing personal and professional development.

### Ethics statement

Ethical approval was granted by the University of Manchester Research Ethics Committee 5 (Reference 2021-8987-17487 and Reference 2021-8987-18579) and The National Research Committee (Reference 2021-258). All participants were provided with an information sheet detailing the purpose of the study, how their data would be used, and how they could withdraw from participation. Informed written consent was obtained from all participants prior to the commencement of their interviews. Due to the sensitive nature of this research, it was extremely important to consider potential distress to the participants and to the researcher. Therefore, a detailed distress protocol was written clearly outlining an explicit process should a participant become distressed. In addition to the protocol, supervision was available to talk through any personal distress and to ensure that the right actions had been taken when participants had experienced distress.

## Results

Six key themes were identified from the data analysis: (1) responsible recruitment, training, and development; (2) dual duty of care; (3) acknowledgement of psychological hardship; (4) superficial support systems; (5) collaborative cultural change; and (6) components of a good model of practice. Sub-themes were developed to highlight important aspects within each theme. While each theme offered rich insights, they also revealed significant interconnectedness, illustrating how toxic masculinity and workplace cultures, combined with insufficient training, support, and supervision, collectively reinforce negative impacts on officer wellbeing. Quotations were selected to reflect prominent patterns in the data, used to underline their truth, and to narrate the participants’ experiences (50).

Throughout the interviews, several officers referred to the concept of opening ‘Pandora’s box’ or a ‘can of worms’ - metaphors used to describe the risk of exposing a multitude of complex issues thought to be contained within prison walls. However, as the interviews unfolded, it became evident that the container had ‘already burst open’. The emotional and psychological difficulties stemming from their work had long since spilled into their personal lives, affecting family relationships, friendships, and home environments. Officers described how these challenges eroded their confidence, morale, self-esteem, and self-worth, severely undermining their wellbeing. Yet, woven through their accounts, often expressed with camaraderie and humour, was a glimmer of hope and a desire to make conditions better for future generations of prison officers and for the prison service.

### Theme 1: responsible recruitment, training, and development

Officers reflected on the impact of staffing changes since 2010, when government-imposed budget cuts led to a significant reduction in prison officers, especially the loss of many experienced officers, resulting in chronic shortages. Ongoing challenges with vetting, recruiting, and training new officers were frequently highlighted. Concerns regarding the increasing number of perceived unsuitable and inexperienced officers being recruited into the service were often expressed. Officers believed these issues are exacerbated by the lack of supportive structures in place such as mentorship and supervision. Furthermore, officers reflected on the lack of both career and personal development opportunities to enhance their skills, progress professionally, and achieve personal growth within their roles. They highlighted the negative impact this has on job satisfaction, morale, and innovation, ultimately weakening organisational performance and staff wellbeing, all of which contribute to high staff turnover. This theme is explored through six interrelated sub-themes: (1) employing and supporting suitable people; (2) realistic preparation and training; (3) mentorship; (4) missed opportunities; (5) professionalism; and (6) career development.

### Sub-theme 1.1: employing and supporting suitable people

Several officers expressed concerns that the current recruitment process is seen as a “quick fix to get boots on landings.” Despite the ongoing chronic shortage of prison officers, the importance of adopting a rigorous recruitment process to ensure suitable candidates are selected for what is widely recognised as a demanding and complex role was strongly emphasised.

Officers felt the role was often misrepresented, with insufficient information given to the emotional, psychological, and relational challenges of the job. They explained how they believed this can lead to the recruitment of unsuitable individuals which they considered both inappropriate and irresponsible.

*“It’s not sold as the right job… it’s painted with a rosy picture… they then struggle and have trouble dealing with it emotionally… a lot are suffering and going off and we do lose a lot of staff through retention”* (Current officer #12, 18 years).

Officers emphasised the importance of thoroughly assessing the overall suitability of applicants prior to recruitment. This was viewed as essential for safeguarding prisoners and maintaining professional standards, as well as protecting the wellbeing of all officers. They explained how they believed the current recruitment process lacks rigour in adequately screening candidates for the maturity and resilience required for the role.

*“In my day you had to be 21… you no longer sit in front of an interview panel, suss you out, nobody interviews you face to face [nowadays], so governors are getting kids turning up to work who do not have clue”* (Current officer #9, 30 years).

The subject of lived experience of mental health difficulties emerged throughout the interviews. Although no officers shared any positive experiences, reflections were made on the potential value of employing individuals with lived experience in helping to reduce stigma and challenge the current macho culture, potentially creating a more psychologically informed environment. However, some officers reflected on the implications of employing people with mental health difficulties. They questioned whether the stressful nature of the prison environment, combined with the demanding role, may exacerbate mental health difficulties, especially in the absence of adequate support systems.

*“They would become more damaged joining the service… and go back into a very dark place. I just think it is irresponsible, it’s just not right, and it is not appropriate… they might have had a period of a couple of years where their mental health stabilised, and they were feeling good and then in this job it will go bad again”* (Current officer #14, 20 years).

One former officer shared how the added layers of responsibilities and pressures on already overstretched officers can lead to feelings of helplessness and frustration when trying to support colleagues with mental health difficulties. They believed the absence of easily accessible professional support services exacerbates this issue and has the potential to create a conflict between endeavouring to support a colleague and meeting the demands of the prison service.

*“So many staff with depression and on antidepressants and there was like a couple of them that I knew that were self-harming… I did not know how to support them”* (Former officer #1, 4 years).

### Sub-theme 1.2: realistic preparation and training

Officers often reported feeling unprepared for the role. They identified contributing factors including insufficient training and support as well as a lack of practical provision, such as not receiving a uniform or safety equipment in a timely manner. This perceived lack of initial preparation adversely affected the confidence of new officers and placed additional strain on existing officers, who were already overstretched, impacting overall safety and operational efficiency.

*“They come back to you on day one, they have got some big gaps in their learning, they have been too busy learning about some theoretical model and administration stuff rather than what I would call the key part of the job in mixing, associating, talking, dealing with prisoners”* (Former officer #2, 32 years).

*“I was stood on the landings… I did not have a uniform… I did not have a radio… I got chucked in and had to teach myself”* (Former officer #10, 4 years).

In addition to a perceived lack of rigor in recruiting, officers identified problems with the current Prison Officer Entry Level Training (POELT). They described how they believed it is not adequate in preparing new prison officers for the realities of prison life and their work with prisoners.

*“It’s very restrictive, all PC [politically correct], you are not allowed to swear, you are not allowed to say how it really is, you cannot be realistic… we are frowned upon if we fail people… but you gotta make people realise what they are going to be dealing with on the landings”* (Current officer #9, 30 years).

Officers reflected on the increasing demands and evolving stresses of their role, emphasising the need for structured support, supervision, and ongoing training. These provisions were seen as essential not only for staying informed about current policies and practices but also for creating space to reflect on their professional roles, process their emotional responses to challenging situations, and adapt to the shifting needs of both prisoners and the wider prison service.

*“I do not think you could ever say that you finished learning as an officer… it’s constantly changing, the security, things change, the drugs change, the needs of the prisoners change, the kind of situations that they are in change… we need to kind of develop with that”* (Current officer #22, 18 months).

### Sub-theme 1.3: mentorship

The importance of having a mentor to learn from and regularly check in with was highlighted as a vital source of support and training for officers throughout their careers. Officers described how mentorship offered ongoing guidance and feedback, enabling them to develop skills in situations as they arose. This support was seen as instrumental in building confidence and competence in their roles. Moreover, effective mentorship was viewed as a key factor in improving staff retention. The value of mentors being experienced officers was strongly emphasised, as their expertise and practical knowledge were considered essential to the effectiveness of the support they provided.

*“I was given a buddy officer who was an older lady who took that role very seriously, took me under her wing taught me everything she knew and if it wasn’t for her, I’d probably left the job a long time ago, but we do not do that anymore”* (Current officer #19, 25 years).

Officers frequently raised their concerns about “the inexperienced leading the inexperienced,” noting that when managers lack adequate experience and training, they may struggle to develop the skills and judgment needed to manage challenging situations, lead effectively, and support their teams.

*“The lack of experience is now coming through into middle management… you just do not have those wise old heads that guide you a bit and look after you a bit”* (Current officer #14, 20 years).

Officers described how “jail craft” is central to their work, highlighting the importance of talking and listening, recognising subtle shifts in the atmosphere on the landings, de-escalating issues before they intensified, and responding swiftly when they did. They explained how this expertise was developed through time and experience in the role. They believed having an experienced mentor who could provide guidance and support was viewed as essential to learning and refining this craft.

*“You use to learn how you deal with tricky situations by copying them [experienced officers]… they have learnt the skills to use their mouth and to talk to prisoners, much better than younger staff who think they are bullet proof… and will inevitably get punched in the mouth within 10 minutes by the prisoner who does not like the way he was talking to them”* (Former officer #5, 23 years).

One officer shared her experience of stepping into a managerial role without mentorship, training, and support.

*“They put me on what was renowned to be the toughest wing in the prison with no managerial experience… there was a huge riot there [reported widely on the news]… I was getting no support, I did not get offered any kind of induction, I did not get any kind of on-the-job training or support, I did not get the opportunity to step back to my old grade or to move to a different wing, I did not even get asked to why it wasn’t working. I was a well-respected member of staff, known to be hard worker… I signed the exit paperwork after 14 years”* (Former officer #27, 14 years).

### Sub-theme 1.4: missed opportunities

Officers reflected on missed opportunities where they believed the prison service could have made better use of their knowledge, insights, and experiences. They explained that failing to do so can result in reduced engagement, motivation, and innovation. They believed that over time, this contributes to low morale which is a major factor in the high turnover of staff.

*“There’s no desire to harness the experience of staff anymore… the experience is down there on the landings… you are gonna find people who have been in the job 30–40 years… no one is interested tapping into that knowledge anymore… we are very much seen as the problem and not the answer”* (Current officer #19, 25 years).

*“They said, oh we know you are only here for another six months… I thought, God, they have already decided that I will not be here rather than looking at what can we do to make you stay”* (Current officer #22, 18 months).

Officers shared how they believed there are also missed opportunities in recognising and rewarding the dedicated service and long-standing commitment of experienced officers, as well as in valuing them as a positive and constructive resource within the prison service.

*“I felt a little bit shunted out [by management], as a bit of a dinosaur… on the morning of my retirement I had 2 minutes with the governor and that was 2 minutes of him talking at me… it could have been much better than that… they could have used us in a much more positive way”* (Former officer #2, 32 years).

An officer also reflected on the lack of recognition and care he received at the end of his career.

*“30 years I give to my country, and they have left me like this [physically and mentally unwell]… 30 years matters… 30 years crown service matters I heard nothing… I wrote to the governor… he wasn’t aware I had left… he said I must have slipped through the net… it was a bullshit answer”* (Former officer #3, 30 years).

An ongoing campaign advocates for the adoption of an earlier retirement age, positioned as both a recognition of prison officers’ service and a means of supporting their physical and mental wellbeing as they transition out of a highly demanding and stressful role.

*“It’s not practical for us to work into our 60’s in such an awful environment… buildings that are dated, plaster falling off walls, full of cockroaches, violent prisoners and their issues and no facilities for staff* ***”*** (Current officer #9, 30 years).

### Sub-theme 1.5: professionalism

Professionalism is regarded as essential to maintaining the legitimacy and integrity of the prison service. Officers described professionalism as encompassing a set of values, behaviours, and relationships that ensure prisoners are treated with fairness and dignity. They emphasised that professionalism extends beyond academic knowledge and technical skills, centring instead on ethical conduct, mutual respect, and a genuine commitment to the responsibilities of the role. Officers highlighted that professionalism fosters trust, not only between officers and prisoners, but also among staff, contributing to a more positive, respectful, and supportive working environment. Ultimately, this was seen as critical to achieving better outcomes for prisoners and upholding the credibility of the prison system.

Officers often described a noticeable decline in respect for the role and observed that professional standards have deteriorated. They believed, in the absence of effective mentorship and supervision, inappropriate or unprofessional behaviour often goes unchallenged.

*“There were strict standards when I joined, minimal make up, no nail polish, very basic jewellery, hair tied back and if you did not you got a good rollicking… whereas now, I look at some of these young girls… they look more dressed up than I do on a night out”* (Current officer #14, 20 years).

*“I see these new members of staff with loads of over familiar touching… there has to be a boundary… to see this physical contact that seems to have developed between staff and prisoners and then these hushed conversations… makes me really sad cause it use to be such a professional service”* (Current officer #19, 25 years).

### Sub-theme 1.6: career development

Career development was identified as a key factor in both professional and personal growth, contributing to improved job satisfaction, higher morale, and overall wellbeing.

*“I have always enjoyed periods of my career when I felt like I had ownership of something… some influence and been able to guide change… without the small wins you get frustrated… if you do not see the impact you have… you do not feel like your cog is that important… and that drains you”* (Current officer #19, 25 years).

Officers frequently reported that a lack of career development opportunities, combined with limited encouragement and support, contributed to heightened stress and frustration. They emphasised the significant emotional, psychological, and physical investment required in their roles and expressed their concern that the evolving demands and responsibilities of their work are not matched by an appropriate wage. As a result, they perceived the role as increasingly unsustainable and, in some cases, intolerable. This was seen to foster a deep sense of dissatisfaction and feeling of being undervalued. Officers believed these factors contribute to high staff turnover, which in turn incurs financial costs related to the recruitment and training of new officers.

*“There was no real career development… staff were not treated very well… as you get a bit older you start thinking what am I doing here? I am giving my health, I’m giving up my sanity and all I am getting back is a paycheck that I can get elsewhere”* (Former officer #27, 14 years).

### Theme 2: dual duty of care

This theme highlights the tension between organisational priorities and resource constraints, illustrating how systemic issues can undermine the prison service’s obligation to provide a safe, humane, and secure environment for both prisoners and staff. Officers reported a perceived imbalance in which their own wellbeing and working conditions were frequently overlooked in favour of prioritising prisoners’ needs and maintaining the regime. This theme is supported by two sub-themes: (1) prisoner-focused culture; and (2) consequences of chronic staff shortages.

### Sub-theme 2.1: prisoner-focused culture

This sub-theme captures officers’ perceptions that institutional priorities are heavily weighted toward running the regime and meeting prisoners’ needs, often at the expense of officer wellbeing. Officers recounted multiple traumatic incidents they had personally endured and shared experiences of how their own needs were overlooked. They described expectations to continue working despite experiencing significant psychological distress. Officers also expressed strong feelings of being treated as expendable commodities. They also reported that assaults against them often went unreported or unsupported to avoid reflecting poorly on the prison’s statistics, whereas incidents involving prisoners were consistently documented and followed by appropriate support. While an emphasis on prisoner care is essential to fulfilling the prison’s mandate, officers felt that this imbalance intensified workplace stress and lowered morale.

*“There was a lifer… he dragged myself and a colleague into a cell and started beating us up… it was awful… He had hold of a knife he wanted to cut us… we had to fight our way out… and there was nothing there for us. I remember seeing the governor go straight to the cell and see how he [the prisoner] was… he went straight to him and took him to the healthcare… to make sure he was ok…and did not say anything to us… just get back to it… I said I need my puffer [angina medication]… he said go on be quick”* (Former officer #3, 30 years).

*“One of my colleagues hit a real mental low… an incident [attempted murder of three staff] affected him so profoundly I do not think he’ll ever recover, it’s been horrific… if this happens for a prisoner then all these boxes have to be ticked… as soon as there’s an incident with three members of staff… it’s not written down anywhere because the managers do not want it on the statistics, they do not want the adjudication statistics to look bad for their prison… so it is hidden and it’s really sad… we are so far down the order of importance when it comes to prison management we are just the lowest of the low”* (Current officer #19, 25 years).

One officer described how access to even basic medical care and support are lacking for prison officers yet are readily available for prisoners.

*“You would get a cut on your hand from something, and you’d go down to the healthcare and ask for a plaster and they say we are not allowed to treat officers just your prisoners”* (Former officer #20, 19 months).

### Sub-theme 2.2: consequences of chronic staff shortages

This sub-theme highlights how systemic issues, particularly chronic understaffing, can profoundly impact the daily experiences and wellbeing of prison officers. Officers voiced serious safety concerns arising from persistent staff shortages, sharing experiences how their own needs and wellbeing were often neglected in the effort to sustain the operational demands of the prison regime. They believed this continual pressure increases stress levels and can leave many officers feeling unsafe, unsupported, and vulnerable, creating conditions that heighten the risk of harm both to staff and the wider prison environment.

*“It’s not just the safety of you, or your colleagues, you just know that when things are stretched, this is when things are just going to slip through and it feels risky”* (Current officer #22, 18 months).

*“You find yourself on your own trying to deal with a difficult situation and there’s hardly ever any consequences for bad behaviour… we do not matter… at which point you walk away… it really knocks your confidence”* (Current officer #14, 20 years).

Officers shared that they were frequently redeployed and how this added further stress and uncertainty to their already demanding roles. As a result, many officers felt undervalued and unimportant, which negatively impacted their confidence and overall wellbeing.

*“They kept cross deploying… I could not cope… it was just so messy, we were just numbers, they were just moving us like we were not really people… it messed up my head and my confidence”* (Current officer #18, 6 years).

Officers described how costly solutions were often used to address staff shortages, as opposed to tackling the underlying causes of poor recruitment and retention.

*“Officers are deployed over 250 miles away… put in overnight accommodation, hotels… because there’s no staff to unlock”* (Current officer #26, 2 years).

A culture of presenteeism, where officers felt compelled to attend work despite being unwell, was frequently described. One officer spoke of the personal sacrifices he made when covering staff shortages and expressed feeling unappreciated and undervalued.

*“It made life hard emotionally, physically, mentally… there were times when I was working 90 plus hours a week because they were short staffed… you do not get any thanks or appreciation… a governor said I need to check whether we are insured for you to come in again, not thanks for coming in”* (Former officer #10, 4 years).

One tragic incident involved a note left by a prison officer. In the note, they described the immense pressure from management to return to work despite being on sick leave.

*“There have been at least half a dozen staff suicides in the last couple of years… one of them left a note and she detailed management pressure to come back to work as she was off sick”* (Current officer #14, 20 years).

### Theme 3: psychological hardship

All prison officers reported experiencing significant psychological hardship stemming from the ongoing demands and stresses inherent in their roles. They felt this hardship often resulted from prolonged exposure to stress and repeated traumatic events, further exacerbated by a perceived lack of organisational care and support. Officers described how these psychological effects extended beyond the workplace, profoundly impacting their personal wellbeing and family relationships. This theme is explored through two sub-themes: (1) psychological distress, trauma, and impact; and (2) spill over.

### Sub-theme 3.1: psychological distress, trauma, and impact

This sub-theme captures the profound emotional and psychological toll experienced by officers, highlighting how daily exposure to stress and trauma can compromise their mental health. It emphasises the urgent need for trauma-informed support systems that recognise, proactively address, and respond to the impacts of prison work, fostering psychological safety and wellbeing while preventing the development of mental ill health.

*“I did not feel well, I spent two weeks where I could not sleep, I could not get out of bed, if I had to get out of bed and was even faced with the thought of putting a uniform on, I was in floods of tears, not just little tears, I was sobbing and that’s not me… I got to the point I could not cope anymore… they offered me nothing, I felt so angry and resentful and poorly… I left… I had a lot of different emotions… forced out for not being good enough… it’s taken me 5 years to realise I did not fail. I was let down”* (Former officer #27, 14 years).

Officers frequently described the emotional labour involved in their roles, where they are expected to regulate and manage their feelings in line with the defined rules and procedures of the prison service.

*“So you are around a dead body for 5,6,7,8 hours because the process that it needs… and at some point you are going to have to go to coroner’s court… but you have still got that guy who took his own life… it could be quite horrific how he has taken his own life… they have what is called a hot debrief… it’s just procedural stuff… Never, and I can say that hand on my heart, never did anyone say after you have dealt with an incident like that, say right ok XXX, or other colleagues, are you ok?”* (Former officer #2, 32 years).

Officers often referred to themselves as “the forgotten service,” hidden behind prison walls and frequently misrepresented or subject to speculation in the media. They explained that this invisibility reinforces toxic masculine and uncaring stereotypes, which not only undermines their morale but also diminishes the social value of their profession and perpetuates inaccurate public perceptions.

*“It’s not a very nice organisation trapped behind a wall… I’ve got PTSD… we just become completely destroyed by it… nobody cares about us… the culture of the media is very much we do not care about prisoners… in fact, a great deal of us, people like me and people I work with that are stood, still care, and treat people with humanity”* (Current officer #17, 17 years).

As a direct consequence of the demanding and stressful nature of their roles, coupled with psychological hardship and trauma, officers described a wide range of impacts on their wellbeing and expressed concern over the perceived lack of adequate support to help them cope effectively.

*“You’ve got a hell of a lot of burnouts now and you are going to have a hell of a lot of mental health issues in the future with nowhere to go and take these issues” (Current officer #14, 20 years).*

Officers reported feeling desensitised as a response to repeated exposures to traumatic incidents. They explained that emotionally distancing themselves serves as a coping mechanism, helping them to get through each day.

*“There’s a level of desensitisation which I think you need… there will be people in the service who will be on the fifth or sixth death and I think they cannot possibly be able to do the job day in, day out without that as a as a coping mechanism”* (Current officer *#22*, 18 months).

Officers described how the perceived lack of care and professional and psychological support to help process traumatic events can have a lasting impact on their wellbeing.

*“I’m so desensitised it’s unreal… this job fucks you up… you do not get any support from you line manager, or senior manager, no one gives a shit… they only care about running a regime. As a human being you are just a fucking number”* (Current officer #9, 30 years).

Officers acknowledged that when they lacked support to process traumatic experiences and faced burnout, their capacity to care for prisoners diminished, often resulting in further emotional desensitisation.

*“I was responding to alarm bells… a woman had made quite a lame attempt of a ligature; I just shook my head and went back into the office… when you become that desensitised it’s dangerous for the women. I knew I had to leave I’d had enough… I’m not sure whether I insisted, or they realised… I think it was me who insisted because they were so desperate for staff, they did not want me to leave”* (Current officer #19, 25 years).

Some officers described a lingering sense of anticipation as they wondered when the accumulative effects of these traumas would surface.

*“I’ve always wondered what effect the job would have on me long term… they say about getting to like a breaking point… resilience is not gonna be indefinite… sometimes I think have I got really good coping skills… or have I not processed it… have I just boxed it? I do not know yet”* (Current officer *#22*, 18 months).

Acknowledging that not everyone copes with or recovers from the psychological toll of their work in the same way is crucial. Several officers shared that they relied on prescription medication to manage the emotional and psychological strain associated with their roles. In contrast, others reported self-medicating with alcohol. This highlights the varying and potentially harmful ways officers attempt to manage the pressures of their work in the absence of adequate support systems.

*“I’ve been on anti-depressants for the last three years… it got really, really difficult and quite stressful… it’s different with mental health it’s not something to see, it’s not like a broken arm… some candles burn out quicker than others”* (Current officer #12, 18 years).

*“I was drinking heavily, I was a fucking physical mess, I was a mental mess… I could not cope”* (Current officer #9, 30 years).

Tragically, some officers do not survive the psychological toll of the job especially without appropriate support.

*“One of the lads I trained with killed himself… he was found hanged in the wing office… he did not receive support… that could be any one of us in the future* (Current officer #16, 8 years).

### Sub-theme 3.2: Spill over

Officers described how psychological distress experienced at work frequently spills over into their personal lives, contributing to work-life conflict. This explained how this not only affects their relationships and overall wellbeing, but it also reduces their ability to recuperate and recharge.

*“It is difficult… the time you get home again you just become the pressures… trying to do the things you want to do is impossible… you are drained all the time”* (Former officer #10, 4 years).

Many officers described the experience of becoming, and being, institutionalised. They reflected on how prison work can shape their social interactions beyond the prison walls.

*“Sometimes you look at yourself and think God I’m institutionalised… your day is so focused on time and regime… your whole day is tracked… that carries over into your personal life, I call people in the shop Mr or Miss… I said to someone at home about something in the next cell, I meant house… it just gets into your brain”* (Current officer #21, 5 years).

Officers often shared how the pressures and psychological toll of their role had a profound and detrimental impact on their home lives and family members, particularly in the absence of appropriate support. In some cases, this led to the breakdown of relationships and family units.

*“I became ratty at home… I did not know I was doing it… I have been suicidal 3 to 5 times and it affects everyone in the family, they have their own mental problems now, seeing me so ill”* (Former officer #4, 32 years).

*“You cannot really understand the continual psychological stress on yourself… and your family… I got up one day, packed my bag and left my wife and daughter”* (Current officer #26, 2 years).

### Theme 4: superficial support systems

Officers perceived current support systems as superficial, offering the appearance of care, but lacking adequate or professional substance. As a result, many felt ill-equipped to manage the emotional and psychological challenges of their work. Several officers characterised these systems as merely “paying lip service,” rather than providing meaningful or effective support.

*“Governors would say you have got a care team available, yeah, but what happens if I do not get on with that member of staff on the care team? Or we do not trust them? Or they cannot be released from their duties?… Then you have got a 24-hours helpline where I’m phoning up some faceless person, I do not know what their qualifications are… so that’s out the window”* (Current officer #9, 30 years).

The prison service was often described as adopting a “tick box approach” investing in resources intended as support but failing to utilise them effectively. They explained current support is frequently led by peers rather than mental health professionals. One officer recounted how, despite receiving training to support colleagues, it was not utilised, which she believed contributed to growing frustration and disappointment among officers.

*“I’ve done the TRiM [trauma risk management] practitioner training… a fantastic asset for staff who have gone through traumatic incidents… I’ve never been asked to do a screening with someone… what is the point of having it in the prison if we are not using it”* (Current officer, 18 months).

Officers often highlighted the need for psychological support to help manage the emotional and psychological impact of their work. However, they explained that such support was neither readily available nor easily accessible.

*“I’ve got images in my head of 6 suicides and an attempted murder… I had planned to end my life… I was on car park roof and was gonna jump off… I rang me family and they got me help… management had not even done the referral [for counselling]”* (Former officer #4, 32 years).

Officers described how they felt their support needs were often trivialised, with psychological help only becoming available once they had reached a crisis point and their mental health had significantly deteriorated.

*“You can ask your manager for 6 one-hour sessions of counselling but it’s not easy to get, you have had to have been off work sick with stress or anxiety to qualify for it”* (Former officer #3, 30 years).

Officers reflected that without accessible and professional support systems, psychological distress can go unnoticed or be misinterpreted, with some masking their emotions and struggling in silence.

*“I have had some of my colleagues commit suicide and one of the tragic things is that we did not identify them, we had no idea, there is a couple I can think of and if we saw them at a social sort of function, you’d think they were the life and soul of the party… if there had been regular support or supervision in place or they had been asked, how are you coping, how are you feeling, what do you need, it could have been helpful”* (Former officer #7, 45 years).

### Theme 5: collaborative cultural change

Collaboratively challenging the existing culture was seen as essential to driving change toward new organisational and occupational values, and fostering a healthier, more supportive prison environment. This theme is explored through two sub-themes: (1) current culture; and (2) shared vision for cultural change.

### Sub-theme 5.1: current culture

The current culture within the prison service is often characterised by pervasive toxic masculinity. Officers described deeply embedded norms and behaviours that promote a harmful version of masculinity, where displays of anger and aggression are common, while emotional expression is mocked or perceived as a sign of weakness or vulnerability, traits that are widely deemed unacceptable. This culture reinforces pressures to conform to hypermasculine behaviours, thereby undermining psychological safety, and discouraging officers from expressing vulnerability or seeking support.

*“I was probably so sucked into that culture that I was guilty of it as well… you laugh and take the piss… you never really thought there could be serious health problems behind it… the care use to be we will go over the road [to the pub] when we finish and have 5 pints, nobody was taking anyone to one side and saying mate are you ok, do you need to speak to anyone, it’s terrible”* (Former officer #27, 14 years).

One officer reflected on the powerful role of group dynamics and stereotypes in sustaining toxic cultural norms. She highlighted how officers can reinforce harmful attitudes and behaviours, even when these conflict with their personal beliefs and values. This potentially contributes to a wider pattern of emotional suppression and dehumanising of officers.

*“Fraggle or fraggled… that’s what they say if a prison officer melts… if he’s gone off ‘cause he saw someone hanging… it’s a pretty normal response to struggle with that… everybody just jumps on that, that sort of culture, that mentality though, but I reckon, if you separated all these officers apart in conversation and said why do you think that they would probably say they do not really know or they do not think that but it’s just when we all get together it’s an awful culture making staff feel sort of ashamed to being a human”* (Current officer #21, 5 years).

Officers are often perceived as embodying stereotypical macho traits such as physical strength. However, bullying within the prison service occurs, causing emotional and psychological distress, often experienced in isolation, particularly when appropriate support systems are lacking, and behaviour goes unchallenged.

*“I’m 6 foot 3 I’m a big guy and I’m the least type of person you would expect to be bullied… I’ve never experienced bullying like that ever and to the point where I would sit in a dark office waiting for people to go home before I would leave”* (Current officer #15, 13 years).

Officers shared their perceptions of the organisational culture as blame oriented and punitive, marked by a lack of concern for officer wellbeing and a reluctance to learn from mistakes or improve existing systems.

*“There’s certainly a blame culture, I think when things go wrong, it’s not right what can we do to look at this? It’s well, who messed up… I think sometimes people get hung out to dry a bit with that”* (Current officer #22, 18 months).

One officer shared how working in a blame culture compounded her trauma, leaving her feeling unsupported and to blame which had long-term psychological consequences.

*“I had a chunk bitten out of my thigh… quite a serious assault… I got back to work within 2 months… I did not get any support… they were pretty much saying it was my fault… I ended up going off with PTSD”* (Current officer #19, 25 years).

### Sub-theme 5.2: shared vision for cultural change

A shared vision for cultural change was identified as essential to addressing the toxic cultures currently prevalent within the prison service. Officers highlighted the importance of adopting a unified set of values, attitudes, and behaviours to cultivate a healthier, more supportive environment. They also emphasised the connection between staff wellbeing and the overall effectiveness of the prison system, advocating for a culture in which officers feel valued, recognised, and supported, ultimately benefiting the entire prison service.

*“A bit of care in the workplace is needed… if you feel so unimportant in the workplace you are gonna actually feel so down and worthless and that is where so many staff are at moment we are just scrapping along the floor as best we can… if we feel valued, we will feel better, and if we feel better prisoners feel better, and that is the natural cycle of a successful prison”* (Current officer #19, 25 years).

Officers emphasised the importance of normalising mental health struggles and fostering emotional openness to create an environment where seeking support during difficult times is accepted and encouraged.

*“Seen as ok to [voice you] have a problem… and chat about it if that’s what you want to do”* (Former officer #2, 32 years).

Officers explained that implementing new support systems must be approached sensitively, with a clear understanding of the existing culture, to be effective.

*“Because of the bravado culture… If you are gonna have a decent support system, it needs to be a bit more clever than someone walking around with a clipboard saying they are the welfare officer… no one is going to talk to you… if it is less public and people can self-refer and just say help me”* (Former officer #27, 14 years).

### Theme 6: components of a good model of practice

Officers reflected on what a comprehensive model of support and supervision within the prison service should entail, identifying key components they considered essential to its effectiveness. This theme is explored through four key sub-themes: (1) acknowledging trauma; (2) promoting good practice; (3) ensuring flexibility; and (4) long-term investment.

### Sub-theme 6.1: acknowledging trauma

Acknowledging trauma was seen as vital to building a supportive workplace culture, where psychological distress is recognised as a natural response to a demanding role. Such recognition can reduce stigma, encourage help-seeking behaviours, and facilitate the provision of appropriate care. Additionally, the importance of compassionate and responsive leadership was emphasised.

*“[For a governor to say] Right, he’s one of our members of staff… I want him rung tomorrow… it does not matter whether he sits and cries down the phone for quarter of an hour… that’s one of our staff and they have been hurt… it never happened though”* (Former officer #1, 4 years).

Officers frequently emphasised the urgent need for proactive support systems that prioritise their wellbeing, especially before crisis points are reached. They also highlighted that the absence of meaningful and effective support contributes to high levels of sickness and staff turnover.

*“We’d lose really good staff because they just were not supported, it was a case of till they go sick or until they go snap, we do not need to act… it’s that kind of culture”* (Former officer #1, 4 years).

The provision for psychological support was emphasised by all officers.

*“When you think about the skills you need in supporting staff psychologically through some really disturbing incidents and essentially you just get a prison officer it’s not good enough”* (Former officer #27, 14 years).

One officer poignantly questioned the absence of mental health screenings within the prison service. Introducing mental health checks alongside the compulsory physical fitness assessments was seen as a potential strategy to reduce stigma and improve care for officers. Such checks would offer officers a safe space to discuss difficulties or concerns and provide a clear pathway to appropriate support services.

*“Why aren’t we given mental health screenings and tests… is our mental health not as important as our physical health?”* (Current officer, #24, 27 years).

### Sub-theme 6.2: promoting good practice

Throughout the interviews, officers described examples of helpful behaviours and approaches, considered good practice within the prison service, that supported their wellbeing.

Officers described a strong sense of care for their colleagues and explained they often relied on one another for support. Camaraderie and dark humour were commonly used as coping mechanisms, particularly in the absence of formal support systems. This peer support was viewed as a natural and continuous form of emotional support.

*“You could have 2 or 3 [suicides] in a few months, and you would again be so reliant on your colleagues… they were the ones that were lifting ya, there was very little, you know, arm around you from above”* (Former officer # #2, 32 years).

Although voluntary support from colleagues can be helpful, it is not always appropriate, particularly due to the deeply ingrained culture of emotional suppression within the prison service, highlighting the need for easily accessible professional support systems to complement peer support.

*“When I joined it was drummed into us that you never show any emotion… you have got your work face and that’s it… it’s very difficult for us to ask for help… you rely on your colleagues… but I could not think of anything worse than crying in front of the male staff in the office, I just could not let myself do it”* (Current officer #14, 20 years).

Officers referenced specialist prison environments, where wellbeing is prioritised, and professional support and supervision are fully integrated into the daily regime. They reflected on the positive impact of these practices in fostering more rehabilitative and supportive cultures for both prisoners and officers.

*“Working alongside psychologists on a personality disorder unit as part of that job we would have group supervision every week… some people naturally just talked about the stresses that they had at home, the stresses that had the last few days at work… you heard the broad spectrum of views”* (Current officer #16, 8 years).

*“I’d like to see more therapeutic communities… if you ever meet a person who’s been through the Grendon process you’ll know straight away because they will talk a different language… so much more self-reflective and that’s the start of rehabilitation for us all”* (Current officer #17, 17 years).

Another example of good practice was the availability of accessible, self-referral services, which contributed to broader benefits across the prison service including improved staff morale, reduced sickness absence, and enhanced staff retention.

*“We had a senior officer who was a trained counsellor… staff would make appointments with him, go in let off steam of the job, about their personal life, about their marriage problems, or debt problems or whatever and the sickness levels plummeted”* (Current officer #9, 30 years).

Officers who received supervision reported increased capacity for self-reflection which enhanced their confidence and professional development.

*“Sometimes supervision can just help you reset… think about what can I actually do… what’s in your control, what do you think would be a good way to go about this or that… running it past your peers… it is just a very supportive space… It’s just a safe space to reflect and learn… you cannot have these discussions on the landings”* (Current officer #22, 18 months).

### Sub-theme 6.3: ensuring flexibility

Flexibility was identified as a key component of a supportive model, allowing for adaptation to officers’ changing needs, accommodating shift patterns and personal responsibilities, and ultimately providing them with greater options and autonomy.

*“It’s not about one size fits all, it’s about meeting the needs of the person not the prison service… a mix of in person and online support and supervision is needed and officers can decide what’s best for them”* (Former officer #3, 30 years).

### Sub-theme 6.4: long-term investment

Officers emphasised the need for sustained financial and cultural investment, advocating for care to be proactively embedded into the core values of the prison service rather than addressed reactively. This approach was viewed as essential for protecting wellbeing, improving officer retention, and ultimately creating a more effective, rehabilitative, and humane prison system.

*“The most obvious thing is if they start investing, they’ll retain better quality staff which means prisoners will get better treatments which then means rehabilitation is more likely”* (Former officer #27, 14 years).

An officer responsible for planning daily regimes explained that with effective management, planning and organisation, incorporating access to structured support, supervision and training was feasible.

*“We can plan it so that would actually physically work, regime is not about how many hours you have got in a day, it’s about what you do with them and it’s about safety and sustainability of that regime, so staff welfare is going to contribute massively to the safety of the regime”* (Current officer #21, 5 years).

Throughout the interviews, officers consistently highlighted the emotional and psychological toll of their work, exacerbated by the perceived inadequacy of existing support systems. They expressed an urgent need for reform to better support them, prevent further harm, and reduce the toxic cultures and environments that continue to pervade the prison service.

*“I’m talking to you tonight [after a long shift] is that I hope with your research… it will stop people getting to where I was wanting to kill myself and still living with PTSD and paranoia”* (Current officer #15, 13 years).

## Discussion

The role of the modern prison officer is increasingly demanding and complex, exacerbated by factors such as overcrowding, rising levels of violence and self-harm among prisoners, chronic understaffing, excessive workloads, and a prevailing culture of toxic masculinity. Interviews conducted in this study, echoed findings from existing literature, revealing a perceived systemic lack of attention and care to recruitment, training, support, and supervision. In the absence of these essential provisions, the pressures of the role have a significant impact on officers’ wellbeing, their personal lives, and the effective functioning of the prison service.

To our knowledge, this study is the first to focus specifically on the experiences of officers in English prisons, examining their perceived need for support, supervision, and wellbeing training. Through reflexive thematic analysis, six interconnected themes were developed: (1) responsible recruitment, training, and development; (2) dual duty of care; (3) acknowledgement of psychological hardship; (4) superficial support systems; (5) collaborative cultural change; and (6) components of a good model of practice. These findings offer new insights into the complex emotional and systemic challenges faced by prison officers, highlighting the urgent need for reform in the how officers are recruited, trained, and supported. They also provide a framework for the design and development of more responsive and effective support structures for prison officers within the prison service. From this, a comprehensive model of care, incorporating robust recruitment practices, adequate training, opportunities for personal and professional development, lifelong learning, and appropriate support and supervision were identified as essential to sustaining what Liebling (19, 52) describes as ‘the moral performance of prisons’.

### Recruitment and training

Prison officers are integral to the effective functioning of the prison service, yet their reflections suggest that current recruitment and training practices fall short in appropriately selecting and adequately preparing individuals for the role. Officers emphasised the need for rigorous recruitment processes, including a realistic portrayal of the job, robust vetting, and interviews, not only to uphold professional standards but also as a strategic response to systemic challenges such as high turnover, operational strain, and deteriorating officer wellbeing. In contrast, poor recruitment practices were seen to fuel a cycle of workforce instability, undermining the service’s ability to retain experienced officers and deliver a safe, rehabilitative regime. Training was also identified as a major concern.

Prison officers criticised the current 6-week POELT programme, describing it as inadequate for preparing new recruits for the emotional and relational complexities of prison life. Consistent with existing literature, participants described an overemphasis on security protocols and the cultivation of a suspicious mindset at the expense of interpersonal and rehabilitative skills, which are central to modern prison work (13, 14, 53). This approach was seen to foster a culture of distrust rather than support. The suspension of the POELT apprenticeship scheme was regarded as a missed opportunity to embed sustained learning, guidance, and structured support for new officers (54).

Aside its potential to build psychological resilience, officers reported that wellbeing training remains largely absent. Integrating such training into the POELT programme and embedding it within ongoing personal and professional development was viewed as vital not only for supporting and sustaining officer wellbeing throughout their careers, but also for enhancing performance and retention.

### Support

Despite the existence of well-developed healthcare systems within prisons and the wider community to support the wellbeing of prisoners, including efforts to better identify and respond to distress (55), officers frequently reported a lack of comparable consideration for their wellbeing needs. This disparity reflects broader systemic issues and inequalities in how the prison system prioritises and responds to the needs of those who live within prisons compared to those who work within them.

Support, in the context of prison officers, refers to the resources and systems designed to help individuals manage the emotional, psychological, and practical demands of their roles. Prison officers’ reflections emphasised the urgent need for multi-layered, easily accessible, and sustainable support structures that extend beyond reactive crisis interventions. They advocated for a continuous and embedded organisational commitment to officer wellbeing and development. This encompassed emotional, psychological, professional, and organisational forms of support, highlighting the importance of a holistic and proactive approach to sustaining workforce wellbeing, resilience, and effectiveness.

### Emotional support

Prison officers described how emotional support was primarily provided through peer networks and informal conversations with trusted colleagues, creating opportunities to share difficult or distressing experiences. While some officers recognised the value of formal initiatives such as peer support schemes, the Care Team, and TRiM, others perceived these as superficial “tick-box” exercises, underutilised, poorly integrated into the prison culture and inconsistently applied. These perceptions may be shaped by entrenched workplace norms that discourage vulnerability and trust, reinforce emotional suppression, and ultimately limit authentic peer connection. Such dynamics are indicative of the emotional labour inherent in prison work (13), wherein staff are expected to manage and suppress their own emotional responses to maintain authority, composure, and professionalism in high-stress, and challenging environments (12). This enforced performance of emotional control was described by officers as both psychologically taxing and isolating, echoing Crawley’s (13) findings that such emotional regulation can have significant personal costs.

Despite these challenges, officers emphasised the importance of fostering a culture in which vulnerability is accepted and encouraged. Such a cultural shift could help normalise emotional responses to traumatic incidents, reduce stigma, and promote psychological safety, key factors in mitigating the harmful effects of emotional labour. Creating psychologically safe environments, such as designated rest areas and informal spaces for emotional decompression, where officers can openly share emotional responses, was seen as essential to normalising trauma responses and easing the burden of emotional labour and sustaining wellbeing. Importantly, officers highlighted the need to support those offering peer support as without proper training, support and supervision, peer supporters may risk emotional exhaustion and secondary trauma. This highlights the need for reciprocal and structured support mechanisms within these initiatives.

### Psychological support

Repeated exposure to traumatic and distressing incidents can have severe and lasting psychological effects (24). Consistent with existing literature, officers frequently described the emotional labour inherent in their roles (13, 22, 34). They also described how the cumulative demands of emotional regulation, especially when unacknowledged or unsupported, had significant and enduring psychological consequences.

Officers strongly advocated for access to self-referral psychological support, which they described as confidential services delivered by qualified mental health professionals to help manage the emotional and psychological toll of their work and address mental health challenges. Rather than limiting support to moments of crisis or significant mental health deterioration, they emphasised the value of early intervention that aligns with their individual schedules and emotional readiness. This reflects broader evidence suggesting that psychological flexibility, described as the capacity to openly engage with difficult thoughts and feelings, can enhance mental health, and build resilience (56–58). Officers expressed a preference for systems that empower them to take ownership of their own wellbeing, including flexible, accessible, and proactive self-referral pathways. Research indicates that when individuals manage their own appointments and determine the frequency and duration of sessions, autonomy increases and waiting times are reduced (59, 60). Therefore, implementing such options within the prison service may help promote autonomy, reduce stigma, and normalise help-seeking behaviours, supporting a cultural shift toward psychological safety and wellbeing.

Prisons can cause considerable harm not only to those incarcerated but also to those who work within them, with effects often extending well beyond the time spent inside the prison environment (61, 62). Reflecting Crawley’s (13) work, many officers described how the pressures of the job frequently spilled over into their personal lives, damaging relationships, contributing to the breakdown of family units, and negatively affecting the wellbeing of those closest to them. These findings highlight the importance of offering dedicated support not only to prison officers but also to their families and loved ones.

Several officers also referred to the concept of a “shelf life,” the belief that prolonged exposure to occupational stress and emotional exhaustion eventually leads to burnout and crisis. This deterioration not only compromises officers’ own health and work performance but also undermines the care, safety, and support provided to prisoners and colleagues. Within prison environments, emotional detachment, often adopted as a coping mechanism, can contribute to desensitised prison cultures in which critical issues such as self-harm, suicidal ideation, or emotional distress are minimised or overlooked (36). Without formal support and supervision structures in place, these challenges can be intensified, leaving officers with limited resources to process the emotional and psychological demands of their work (21).

Several officers shared personal experiences of losing colleagues to suicide, an issue with profound individual and systemic implications. Suicide is a significant public health concern (63), with long-lasting consequences for families, friends, and co-workers (64). In 2022, there were 5,642 registered suicides in England and Wales (65). Prisoner suicide rates within prisons are considerably higher than in the general population, with suicide being the leading cause of preventable death in these settings (66). Recent research has explored potential risk factors for suicide and self-harm among prisoners (67, 77), as well as officers’ attitudes and experiences regarding prisoners who self-harm (68, 69). However, to our knowledge, there have been no formal studies capturing the prevalence or rates of suicide and self-harm among prison officers in the UK. International data suggests correctional officers are at elevated risk and have higher suicide rates compared to the general population (70). This critical issue demands further investigation and highlights the urgent need for appropriate support services not only for officers but also for their families and significant others.

### Professional support

Professional support was understood to include structured mentorship, supervision, and reflective practice, typically facilitated by experienced practitioners. These forms of support were viewed as essential to officers’ ongoing development, emotional resilience, and ability to navigate the complexities of prison work.

Mentorship was identified as a crucial, yet inconsistently available, form of support that significantly influenced officers’ career longevity, professional development, and personal growth. Several officers described mentorship as a key factor in their decision to remain in the service, emphasising its role in navigating occupational culture, building confidence, and managing the emotional demands of the role. Effective mentorship can also enable the cultivation of ‘jail craft’, the intuitive, experience-based skills necessary for maintaining safety, managing relationships, and upholding institutional order (18). These findings suggest that embedding structured formalised mentorship programmes across all career stages could enhance individual resilience and competence while contributing to the overall stability and professionalism of the prison workforce.

Officers who engaged in supervision and reflective practice reported significant benefits in managing complex and emotionally charged situations. Notably, these positive outcomes were most evident in specialist settings such as TCs, PIPEs, and the UG scheme, where regular, mandatory, structured supervision was facilitated by trained supervisors. Officers described how these sessions created a psychologically safe environment that enabled them to critically reflect on their practice, process emotional responses, and receive constructive feedback. This approach aligns with clinical supervision models common in healthcare, emphasising professional accountability, emotional resilience, and the development of psychological insight. Expanding the implementation of such structured supervision and reflective practices across the wider prison estate could substantially enhance professional standards and improve overall prison culture. When consistently applied at both individual and group levels, these practices have the potential to cultivate a workforce characterised by greater emotional intelligence and resilience, thereby supporting staff wellbeing, and promoting the delivery of a rehabilitative, high-quality prison service (43).

### Organisational support

Organisational support was identified as critical in mitigating the pressures faced by prison officers. While prison overcrowding is primarily a systemic issue driven by broader government policies and societal factors, the prison service plays a crucial role in managing its impacts. This includes adapting operational practices, ensuring safety, and advocating for necessary resources, particularly by maintaining adequate staffing levels to reduce workload burdens, prevent burnout, and sustain operational effectiveness (5). Officers described policies that prioritise their wellbeing, such as access to rest spaces, especially after exposure to traumatic incidents, are essential to safeguarding their wellbeing. Importantly, the prison service must recognise its dual duty of care by acknowledging both the physical and psychological demands placed on officers. Fulfilling this responsibility and recognising the intrinsic link between officer wellbeing and institutional effectiveness is essential. This requires validating the emotional labour inherent in officers’ roles, explicitly addressing trauma and psychological hardship, and ensuring access to high-quality training, structured supervision, and ongoing support. By embedding these practices into organisational culture, the prison service can foster a healthier and more resilient workforce that is better equipped to navigate the emotional and operational complexities of the prison work.

### Collaborative culture change

Officers described a prevailing culture of toxic masculinity within prisons, highlighting the urgent need for collaborative cultural change to foster healthier, more supportive working environments. As described by officers, this cultural dynamic often creates a conflict between individual values and dominant institutional norms, revealing deep tensions that affect staff wellbeing and behaviour. This aligns with Munsch et al. (71), who found that individuals in Masculinity Contest Cultures (MCC) often mistakenly believe their peers fully endorse such norms, even when many do not. Recent research into prison governors’ wellbeing identified MCC as characterised by organisational norms that promote competition, work devotion, emotional suppression, strength, and dominance, factors shown to undermine wellbeing (41). The sustained reinforcement of these norms has contributed to the emergence of a more extreme variant, hyper-MCC (41). Conversely, positive organisational cultures are strongly linked to better experiences and outcomes for prisoners and prison officers (16, 40). Importantly, creating supportive prison cultures is a collective responsibility, with everyone playing a role in either reinforcing or challenging prevailing norms (72). Through intentional collaborative efforts to challenge toxic masculinity, supporting reflective practice, and promoting continuous learning, meaningful cultural change is achievable, with benefits extending beyond staff to prisoners and the wider community.

### Strengths and limitations

This research contributes to the literature by highlighting the emotional labour inherent in prison officers’ work, the psychological impact they experience, and by identifying their support, supervision, and training needs.

A key strength of this study is the inclusion of both former and current prison officers, including one participant from the Unlocked Graduates scheme. This contributed to a rich and diverse range of perspectives. Another notable strength is the use of RTA, which enabled an in-depth and nuanced exploration of officers’ lived experiences. This method is inherently subjective and interpretative, relying on the researcher’s active engagement with the data and ongoing reflexivity throughout the analysis process (50). As with most qualitative research, the goal was not to generalise findings but to provide a contextualised and meaningful understanding of complex experiences (73). However, these findings offer valuable insights and may inform broader policy, practice, and cultural change within the prison service.

This study has several limitations. Although the initial aim was to recruit prison officers from both England and Wales, all participants were from English prison environments. Including perspectives from officers across the wider UK prison estate would have provided a more representative and comprehensive understanding of the issues explored.

Participant recruitment was conducted through the distribution of a flyer to POA members via POA communication channels. While the response rate was lower than anticipated, this is not uncommon in research involving frontline staff such as prison officers, whose demanding schedules and limited availability can constrain participation. Nevertheless, the 27 interviews conducted offered valuable insights and reflected a broad range of experiences and perspectives.

Data was not specifically collected on the number of prisons represented or the officers’ grades or positions. Therefore, it is not possible to report how many prisons were involved or the specific roles of participating officers. However, the interviews revealed that participants had worked across a range of prison establishments, spanning various security categories and operational functions, indicating a breadth of experience and perspectives within the sample.

The sample was predominantly male and entirely white. While this aligns with the current demographic profile of prison officers in England and Wales, who are predominantly male and predominately white (30), it limits the diversity of perspectives captured.

The demographic composition of this study may have influenced the themes identified, potentially overlooking critical issues related to inclusion, identity, and discrimination, factors that can significantly impact the working lives and wellbeing of minoritised officers. The absence of voices from underrepresented groups, such as officers from ethnically diverse backgrounds and those identifying as LGBTQ+, means that the unique experiences and specific support needs of these individuals may not have been fully captured.

No comparative analysis was conducted between current and former officers, as it is beyond the focus of this study. This study aimed to identify common themes in support, supervision, and wellbeing training needs across the workforce, rather than differences based on employment status. However, this distinction may offer useful insights for future analysis. Similarly, gender was not analysed as a variable in this study, as the primary focus was on systemic challenges and resource gaps experienced by prison officers more broadly. However, gender remains a relevant factor and warrants further investigation in future work.

## Conclusion

Wellbeing is increasingly recognised in government policy as a critical factor in improving quality of life and promoting public health (74). However, this recognition is not consistently extended to prison officers, whose roles involve significant emotional labour, psychological strain, and exposure to trauma.

The prison service holds a duty of care to provide a safe, humane, and secure environment, not only for prisoners but for officers. When prison officers are left unsupported, the consequences extend beyond the prison walls, affecting their mental health, personal relationships, and the wider communities. Suicide among prison officers is a pressing concern, highlighting the urgent need to address toxic workplace cultures and prioritising officer wellbeing. As such, prison officer wellbeing should be understood not only as an operational concern, but as a critical public health issue.

Addressing these needs requires the implementation of flexible, accessible, and embedded psychological support and supervision systems. Investment in a comprehensive and consistent model of care, adaptable to the evolving needs of officers, is essential. Such investment would help to strengthen resilience, improve retention, and foster a healthier prison environment. Ultimately, prioritising officer wellbeing represents a crucial step toward humanising the prison service and ensuring its long-term ethical and operational sustainability.

## Data Availability

The datasets generated during this study are not publicly available due to ethical considerations, including the need to protect participant confidentiality and ensure methodological integrity. Further queries can be directed to the corresponding author.
