# Correction: Reflecting the voices of prison officers with respect to their support, supervision, and wellbeing training needs: a reflexive thematic analysis

**DOI:** 10.3389/fpubh.2025.1712944

**Published:** 2025-11-12

**Authors:** Frontiers Production Office

**Affiliations:** Frontiers Media SA, Lausanne, Switzerland

**Keywords:** prison officer wellbeing, prison officer support, prison officer supervision, prison cultures, prison officer culture, emotional labour

Due to a production error, the published article did not incorporate the corrections submitted by the authors. This notice serves to rectify those omissions.

A correction has been made to the following sections:

In the **Introduction** section, paragraph 8, the sentence “Additionally, prolonged occupational stress, exacerbated by heavy workloads, long working hours, and role conflict, where officers must balance security with care (20), contributes to emotional exhaustion, burnout, poor morale, reduced job satisfaction, absenteeism, and high staff turnover (27–29).” was corrected to “Prolonged occupational stress, intensified by heavy workloads, long working hours, and role conflict, where officers must balance security with care (20, 79), can contribute to emotional exhaustion, burnout, low morale, reduced job satisfaction, absenteeism, and high staff turnover (27, 29).”

In paragraph 9, the sentence “This is further exacerbated by the presence of toxic masculinity, which intensifies the emotional demands required of prison officers, often leading them to adopt a façade of coping while suppressing genuine emotional responses (38).” was corrected to “This is further exacerbated by the presence of toxic masculinity, which intensifies the emotional demands required of prison officers, which may lead them to adopt a façade of coping while suppressing genuine emotional responses (38).”

On page three, paragraph 3, the sentence “A systematic review of the literature identifying support and supervision for prison officers found that support is predominantly provided informally by peers (22).” was corrected to “A systematic review of the literature, investigating the support and supervision needs of prison officers, was reported as an empty systematic review as it found no evidence that met the inclusion and exclusion criteria and the protocol registered with PROSPERO (22).”

In the **Results** section paragraph 1 The sentence “Six key themes were constructed from the data analysis: (1) responsible recruitment, training, and development (2) dual duty of care (3) acknowledgement of psychological hardship (4) superficial support systems (5) collaborative cultural change (6) components of a good model of practice.” was corrected to “Six key themes were identified from the data analysis: (1) responsible recruitment, training, and development; (2) dual duty of care; (3) acknowledgement of psychological hardship; (4) superficial support systems; (5) collaborative cultural change; and (6) components of a good model of practice.”

In subsection “***Theme 6: components of a good model of practice***,” paragraph 1, the sentence “Officers reflected on what a comprehensive model of care within the prison service should entail, identifying key components they felt were essential to its effectiveness.” was corrected to “Officers reflected on what a comprehensive model of support and supervision within the prison service should entail, identifying key components they considered essential to its effectiveness.”

In the **Discussion** section, paragraph 1 the sentence “The role of the modern prison officer is increasingly demanding and complex, compounded by overcrowding, chronic understaffing, excessive workloads, and a prevailing culture of toxic masculinity.” was corrected to “The role of the modern prison officer is increasingly demanding and complex, exacerbated by factors such as overcrowding, rising levels of violence and self-harm among prisoners, chronic understaffing, excessive workloads, and a prevailing culture of toxic masculinity.”

In paragraph 2 the sentence “They also provide a framework for the design and development of more responsive and effective support structures within the prison service.” was corrected to “They also provide a framework for the design and development of more responsive and effective support structures for prison officers within the prison service.”

In the subsection “***Recruitment and training***” paragraph 2, the sentence “Officers criticised the current POELT programme, which has been reduced to just six weeks, as inadequate for preparing recruits for the emotional and relational complexities of prison life.” was corrected to “Prison officers criticised the current 6-week POELT programme, describing it as inadequate for preparing new recruits for the emotional and relational complexities of prison life.”

In the subsection “***Emotional Support***” paragraph one, the sentence “These perceptions could be shaped by entrenched workplace norms that discourage vulnerability and trust and reinforce emotional suppression which may limit authentic peer connection.” was corrected to “These perceptions may be shaped by entrenched workplace norms that discourage vulnerability and trust, reinforce emotional suppression, and ultimately limit authentic peer connection.”

In the same paragraph the sentence “This enforced performance of emotional control was described by officers as both psychologically taxing and isolating, echoing Crawley (18) observation that such emotional regulation can have significant personal costs.” was corrected to “This enforced performance of emotional control was described by officers as both psychologically taxing and isolating, echoing Crawley's (18) findings that such emotional regulation can have significant personal costs.”

In the subsection “***Psychological support***” paragraph 4, at the end of the sentence “Without formal support and supervision structures in place, these challenges can be intensified, leaving officers with limited resources to process the emotional and psychological demands of their work,” reference 38 was removed.

In paragraph 5, the sentence “Suicide rates within prisons are considerably higher than in the general population, with suicide being the leading cause of preventable death in these settings (67).” was corrected to “Prisoner suicide rates within prisons are considerably higher than in the general population, with suicide being the leading cause of preventable death in these settings (67).”

In the subsection “***Professional support***” paragraph 2 the sentence “Effective mentorship enabled the cultivation of ‘jail craft', the intuitive, experience-based skills necessary for maintaining safety, managing relationships, and upholding institutional order (19).” was corrected to “Effective mentorship can also enable the cultivation of ‘jail craft', the intuitive, experience-based skills necessary for maintaining safety, managing relationships, and upholding institutional order (19).”

In paragraph 3 the sentence “Notably, these positive outcomes were most evident in specialist settings such as TCs, PIPEs, and the UG scheme, where regular, structured supervision was facilitated by trained supervisors.” was corrected to “Notably, these positive outcomes were most evident in specialist settings such as TCs, PIPEs, and the UG scheme, where regular, mandatory, structured supervision was facilitated by trained supervisors.”

Reference 79 has been added: Liebling A, Price D, Shefer G. *The Prison Officer*, 2nd Edn. London: Willan (2010). doi: 10.4324/9780203832998”

Reference 33 was corrected from “Kinman G, Wray S, Strange C. Emotional labour, compassion fatigue and wellbeing among prison nurses. *J Forensic Nurs*. (2016) 12:123–32. doi: 10.1097/JFN.0000000000000115” to “Kinman G, James Clements A, Hart J. Work-related wellbeing in UK prison officers: a benchmarking approach. *Int J Workplace Health Manag*. (2016) 9:290–307. doi: 10.1108/ijwhm-09-2015-0054”

Reference 34 was corrected from “Kinman G, James Clements A and Hart J. Work-related wellbeing in UK prison officers: a benchmarking approach. J. Workplace Health Manag. (2016) 9:290–307. doi: 10.1108/ijwhm-09-2015-0054” to “Kinman G, Clements A. *POA Survey of Work-related Wellbeing 2020*. London: Prison Officer Association (2020). doi: 10.13140/RG.2.2.20527.87203”

Reference 35 was corrected from “Kinman G, Clements A. POA Survey of Work-related Wellbeing 2020. (2020). doi: 10.13140/RG.2.2.20527.87203” to “Nylander PÅ, Bruhn A. The emotional labour of prison work. In: Phillips J, Waters J, Westaby C, editors. *Emotional Labour in Criminal Justice and Criminology*. London: Routledge (2020). p. 69–84.”

References 13 and 18 have been merged. Reference 13 has now been updated to “Crawley E. Doing prison work: The public and private lives of prison officers. Devon: Willan (2004).” and all other references have been renumbered accordingly.

The original version of this article has been updated.

